# Deep Learning–Based Methods for Automatic Diagnosis of Skin Lesions †

**DOI:** 10.3390/s20061753

**Published:** 2020-03-21

**Authors:** Hassan El-Khatib, Dan Popescu, Loretta Ichim

**Affiliations:** Faculty of Automatic Control and Computers, University Politehnica of Bucharest, 060042 Bucharest, Romania; service2@proton.com.ro (H.E.-K.); loretta.ichim@upb.ro (L.I.)

**Keywords:** image processing, deep learning, machine learning, convolutional neural network, artificial intelligence, skin lesion detection, neural network, dermoscopic image

## Abstract

The main purpose of the study was to develop a high accuracy system able to diagnose skin lesions using deep learning–based methods. We propose a new decision system based on multiple classifiers like neural networks and feature–based methods. Each classifier (method) gives the final decision system a certain weight, depending on the calculated accuracy, helping the system make a better decision. First, we created a neural network (NN) that can differentiate melanoma from benign nevus. The NN architecture is analyzed by evaluating it during the training process. Some biostatistic parameters, such as accuracy, specificity, sensitivity, and Dice coefficient are calculated. Then, we developed three other methods based on convolutional neural networks (CNNs). The CNNs were pre-trained using large ImageNet and Places365 databases. GoogleNet, ResNet-101, and NasNet-Large, were used in the enumeration order. CNN architectures were fine-tuned in order to distinguish the different types of skin lesions using transfer learning. The accuracies of the classifications were determined. The last proposed method uses the classical method of image object detection, more precisely, the one in which some features are extracted from the images, followed by the classification step. In this case, the classification was done by using a support vector machine. Just as in the first method, the sensitivity, specificity, Dice similarity coefficient and accuracy are determined. A comparison of the obtained results from all the methods is then done. As mentioned above, the novelty of this paper is the integration of these methods in a global fusion-based decision system that uses the results obtained by each individual method to establish the fusion weights. The results obtained by carrying out the experiments on two different free databases shows that the proposed system offers higher accuracy results.

## 1. Introduction

Melanoma is a type of skin cancer considered one of the deadliest forms of cutaneous cancer [1], being able to metastasize very fast. According to the statistical data presented by the World Health Organization and the International Agency for Research on Cancer, through the project Globocan, the global incidence of melanoma is growing steadily [2]. In Romania, 25-30% of patients are diagnosed in advanced stages, III and IV [3]. According to the 2019 annual report of the American Cancer Society, it was estimated that there will be approximately 96,480 new cases of melanoma and 7230 people will die from the disease [4].

Melanoma usually appears as an irregular mole. Melanoma can develop on an existing mole that has changed, on a newly formed mole, but it can also appear on another skin sign, or on a skin portion without any sign. More advanced lesions may display inflammation, ulceration, itching or bleeding. However, some melanomas do not have the typical color of a mole. They can also be smaller than 5 mm, while moles are typically larger than 5 mm. They can occur in areas that are never exposed to the sun. In this moment the diagnosis is performed by rigorous local examination of the lesion by a dermatologist. Suspicion of malignant melanoma is elevated by tumors with the following aspects: irregular surface, rapid growth in size, asymmetrical, with differentiated pigmentation varying from brown to black (with violet tint), and the presence of hyperpigmentation islands. The diagnosis of certainty is made by anatomical-pathological examination of the excised tumor. The problem is that the extraction of the lesion is an invasive method. However, if detected and removed on time, more than 90% of melanoma cases are curable. If the disease is identified and treated late, when there are already liver or lung tumor metastases, the survival rate after surgery drops well below 20%. Therefore, a non-invasive computer–aided system that can help patients directly detect the melanoma, is necessary.

Over the time, different researchers have tried to create a skin lesion detection system using deep learning and machine learning techniques. For example, the authors in [5] developed a skin lesion classification system whose first step was a preprocessing one that consists of data augmentation. This operation was followed by the feature extraction step using a pre-trained AlexNet convolutional neural network (CNN). Finally, the decision step was realized by using a K-nearest neighbor (KNN) algorithm. The results obtained on a database of 399 images showed high biostatistic parameters values. The algorithm had a specificity of 95.18%, a sensitivity of 92.1% and an accuracy of 93.64%. Codella et al. [6], also used an AlexNet CNN in order to extract some features. This paper used one of the best-known image databases dedicated to skin lesion detection, namely the International Skin Imaging Collaboration (ISIC) database, which consists of 2624 dermoscopic images including melanoma and non-melanoma nevi images. The AlexNet CNN outputs where adapted, and they used features from sparse coding, a low-level handcrafted feature and a deep residual network. In order to classify the images, a support vector machine was used. The accuracy was 93.1%, specificity 92.8% and sensitivity 94.9%. 

More types of skin lesions (12 types) were classified in [7] based on ResNet-152 CNN. The results obtained on 956 images were framed between the following values: 89% in case of warts and 96% in the case of acnitic keratosis. Another work that addressed the topic of skin lesion classification with multiple CNNs was [7], in which the authors compared the results given by three kinds of CNN: residual networks (ResNet), VGG19, and the hybrid of VGG19 with the support vector machine (SVM). The database contained 10000 images of benign and malignant lesions. The best result was obtained using VGG19, the sensitivity reaching a value of 95% [8]. Kawahara et al. [9] demonstrated the high accuracy of a linear classifier, which is trained on features extracted on a CNN pre-trained on natural images. The system could successfully differentiate up to ten types of skin lesions.

Some authors [10] turned their attention to the accurate segmentation of skin lesions. The technique applied in this paper is also related to the deep learning approach. The first step was to reduce the noise on the image by applying some filters and then, the image resulting from this preprocessing step was subjected to a CNN. The obtained results where good, with a sensitivity of 95%, specificity of 98.9% and an accuracy of 98.5%, but only for segmentation, not for classification. More recently, a new method for skin lesion segmentation was presented in [11], where a dense deconvolutional network is trained for keeping the resolution of input and output images without any difficult postprocessing of the images. A chained residual pooling is then used in order to extract contextual information. To obtain a good prediction mask, a hierarchical supervision is used. An accuracy of 0.939%, a Jaccard index of 0.756% and a Dice coefficient of 0.866% are then obtained. The algorithm was applied on ISBI 2017 database. In [12], besides classic features, like geometric features, the authors used the histogram of oriented gradients (HOG) and texture descriptors such as fractal dimensions in order to detect the skin lesions. The fractal analysis showed the best results in classifying the skin lesions. 

Milton [13] used deep learning models such as InceptionResNetV2, InceptionV4, PNASNet-5-Large and SENet154 on the ISIC 2018 database in order to classify skin lesions, the best obtained result being a validation score of 76% obtained by using PNASNet-5-Large.

End-to-end learning was also used in some studies by using a pre-trained CNN. Esteva et al. [14], used the GoogLeNet Inception v3 CNN in order to classify the skin lesions. The pretraining of the CNN was done by using the ImageNet database. The fine-tuning was then done by using transfer learning in order to distinguish cancerous lesions from benign lesions.

A fully convolutional neural network, called DermoNet is proposed by the authors in [15]. This is a network in which the layers can reuse the data from the previous layer. This paper uses some databases including the database used by us in this paper (PH^2^ database). The result in case of PH^2^ database is 85.3% Jaccard coefficient [15]. An analysis of skin lesions aimed at melanoma detection is also done in [16]. In order to address lesion segmentation, feature extraction and classification of lesions two deep learning methods are proposed (two fully convolutional residual networks – FCRN). The obtained results show an accuracy of 0.753 for lesion segmentation, 0.848 for feature extraction and 0.912 for image classification.

In this paper we proposed a new decision system that combines several machine learning and deep learning methods, so we can automatically recognize the skin lesions with melanoma appearance with a high accuracy. For our experimental results on two databases, PH2 and ISIC 2019, the accuracy was about 93%–95%. The images from both the PH2 and ISIC 2019 databases have associated the true diagnosis based the clinical diagnosis and on histopathological test. One neural network, three other CNNs and a feature-based methods are used in order to distinguish the common nevus from melanoma. To achieve highest accuracy, the system uses the accuracies obtained by each method separately. Depending on the accuracies obtained, each method contributes with a certain percentage to the final decision given by the main system. In the CNN case, the GoogleNet, residual neural network (ResNet)-101 and neural architecture search (NasNet)-Large were fine-tuned by using transfer learning are used in order to detect the skin lesions. Regarding the feature-based method that helps us on the automatic recognition of skin cancer, the classification is done by using a support vector machine (SVM). The main authors’ contributions consist in proposing a new neural network and decision fusion of multiple neural networks based on associated weights to melanoma detection.

The rest of this paper is organized as follows: Section 2 provides a summary of the databases and data preparation used in the experiments and, also, different methodology for skin lesion detection. Section 3 describes the experimental results. Discussion of the proposed methods and comparisons with other works in the domain are given in Section 4. Finally, Section 5 concludes the paper.

## 2. Materials and Methods

First, we describe the used databases and then we detail the proposed methodology to detect skin lesions. All the presented algorithms have been run on a computer equipped with a 4.5 GHz (turbo boost) Core I7, 9^th^ generation Intel processor, and 16 GB RAM.

### 2.1. Database and Data Preparation

All the images used in this paper in order to train and test the proposed algorithms are images extracted from two different free dermoscopic images databases: the PH^2^ database [17] and the International Skin Imaging Collaboration (ISIC) 2019 database [18,19,20].

In the case of the PH^2^ database, the clinical diagnosis of the dermoscopic images was made by dermatology experts analyzing several dermoscopic criteria such as: pigment network, dots/globules, streaks dots/globules, colors regression areas, and blue-whitish veil [17,21]. The PH^2^ database has been specially developed for research purposes, to facilitate the studies regarding comparations on both classification and segmentation and algorithms of skin lesions images. The database was acquired at the Dermatology department of Hospital Pedro Hispano (Matosinhos, Portugal). The images were acquired using a magnification of ×20 under the same conditions as Tuebinger Mole Analyzer system [17]. The database consists of 200 dermoscopic images of melanocytic lesions. Of these, 80 images represent common nevi, 80 atypical nevi, and 40 melanomas. All the images are labeled with the diagnostic information. In this paper, we selected 60 images of common nevus and 40 melanoma images from the PH^2^ database in order to train and validate the proposed algorithms. Even though the images contained in the database are not just clear images, as the database also containing skin lesions covered by hair, a lot of studies have shown good results when applying their algorithms on this database. According to [17,22], the database contains images having a resolution of 768 × 560 pixels. 

**Figure 1:**
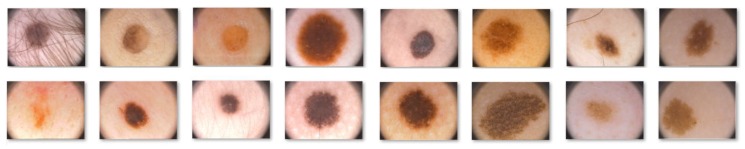
Common nevus images extracted from PH^2^ and ISIC 2019 databases.

**Figure 2:**
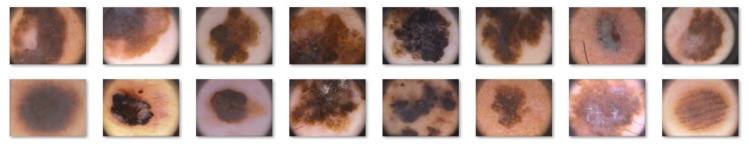
Melanoma images extracted from the PH^2^ and ISIC 2019 databases.

The ISIC is a collection of some databases including 25,332 JPEG images of skin lesions. Basically, the archive is made up of three large databases, namely, BCN_20000, HAM 10000, and MSK. The large image database contains the most common classes of skin lesions including, nevi, vascular lesions, squamous cell carcinoma, dermatofibroma, melanoma, actinic keratosis, basal cell carcinoma, solar lentigo and seborrheic keratosis [18,19,20]. As in the PH^2^ database, not all the images are clear, the presence of hair creating problems from this point of view. Like the PH^2^ database, the ISIC 2019 was specially developed for research purposes in order to support the development of new algorithms that can help in the automatic detection of melanoma. We selected from ISIC 2019 double the number of images than from the PH^2^ database: 80 melanoma and 120 common nevus images. Examples of images from the both the PH^2^ and ISIC 2019 databases are shown below (Figure 1 and Figure 2, respectively).

As mentioned above, an image preprocessing consisting in hair removal is need before applying the proposed algorithms. The problem of algorithms for hair removal from skin lesions is a long-standing topic. Many studies have been carried out on this subject and most of them presented really good results. One of these studies was carried out by the authors in [23]. They proposed a pre-processing software called DullRazor. The first step of this algorithm consists of identification of the dark hair locations by a generalized grayscale morphological closing operation. Then, the shape of hair pixels is verified considering thin or long structure. These pixels are then replaced using a bilinear interpolation. Finally, an adaptive median filter is used to smooth the replaced pixels. As the authors mentioned, the algorithm showed good results in most images excepting the images where the hair was very thin and where the very thick hair covers the skin lesion. In this case, a part of the skin lesion was also removed together with the hair, leaving traces on the lesion [23]. Like a hair detector, DullRazor uses generalized morphological closing which is a simple and efficient one. There are also others hair detectors like: Prewitt edge detector, top-hat operator, multiscale matched filters, derivative of Gaussian, multiscale skeletons and morphological operators, etc.

**Figure 3:**
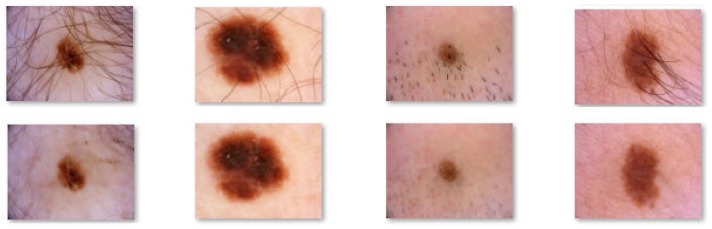
Results obtained after applying the DullRazor algorithm on common nevus images.

**Figure 4:**
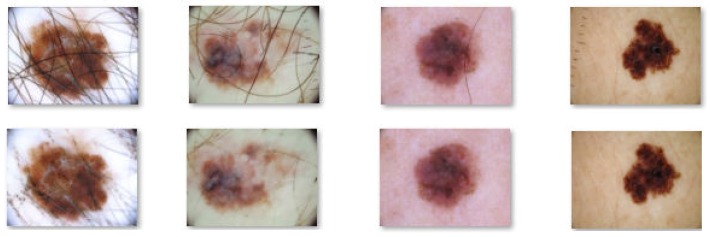
Results obtained after applying the DullRazor algorithm on melanoma images.

In this paper, in order to remove the hair from the skin lesion images we also used the DullRazor algorithm. Figure 3 and Figure 4 shows the results obtained by applying DullRazor to our selected images.

As can be seen from the above images, in both melanoma and common nevus cases the results after applying the DullRazor algorithm are good, except for the images where very thick hairs cross the lesions, such as in the first image on the left side in Figure 4, where a few hairs couldn’t be completely removed. Also, in Figure 4 we can see, on the image placed on the right side, that the algorithm also helps us to eliminate certain signs and markers made by the dermatologist near the lesion, signs that can be mistaken with skin lesion or hair and can lead to poor results.

### 2.2. Methodology for Skin Lesion Detection Using the Proposed Neural Network

**Figure 5:**
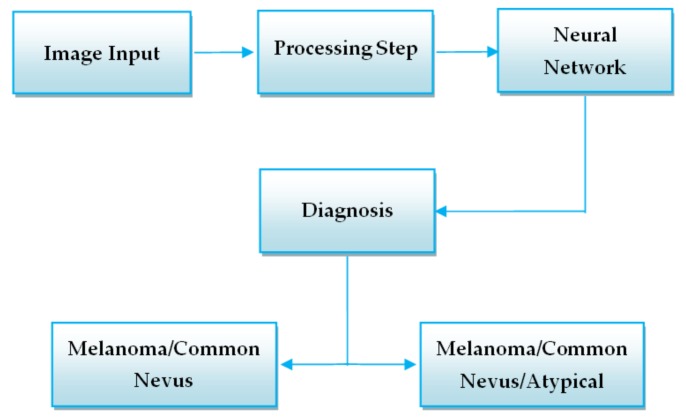
Proposed method – Neural Network used as classifier.

In order to develop a high-performance system that could correctly distinguish melanoma from other lesions we need first to preprocess the images (Figure 5 – Preprocessing Step).

Then, the image is resized so to obtain a uniform representation and the image gray-scale conversion represents the preprocessing step. The next step is the classification using a neural network. First it is necessary to build the training matrix and the output matrix and after that, we need to build some hidden layers. Finally, the classification is performed. The system has an output layer consisted from two targets, melanoma and common nevus.

In order to implement the algorithm, the MATLAB software was used. Functions such as the “network” function and “nntraintool” were used. The function “nntraintool”, causes the appearance of the training GUI, but this happens just before the training is finished. Details regarding the training algorithms such as network accuracy and some useful plots can be seen in the training windows. “Patternnet” matlab function was also used in order to create the neural network. This function gives a two-layer-feed-forward network using sigmoid hidden and softmax output neurons. When given enough neurons in the hidden layer it can classify vectors very well.

Equation (1) describes the working process of a neural network [24].

(1) $$a_{j}^{l}=\sigma \left(\underset{k}{\sum }w_{jk}^{l}a_{k}^{l-1}+b_{j}^{l}\right)$$

The above sum is over the neurons *k* in the layer (*l*−1). The weight matrix for each layer *l*, is noted with *w^l^*; *j* and *k* represent de row and, respectively, the column. For each layer *l* the bias vector, *b^l^* is defined. The bias vector components are the values *b^l^_j_*, one component for each neuron in the *l* layer. The activation vector is noted with *a^l^* which components *a^l^_j_* are the activations [25].

The training is performed using scale conjugate gradient backpropagation. More precisely, this function takes a raw of *N* hidden layers sizes and a backpropagation training function and returns an *N*+1layer pattern recognition network. The input and the output layer sizes are set to zero. This size can be manually or automatically configured to match data by “train function”. This last function is also used to train the neural network. It takes a network, an input data, and a target data and returns the training [24].

### 2.3. Methodology for Skin Lesion Detection Using Convolutional Neural Networks

The networks trained on a big number of images in order to classify other images in a large range of objects are named pre-trained image classification networks [26]. Thus, the feature representations of many images were learned by the networks. The other three methods that we proposed in order to detect skin lesions uses CNN’s that are pre-trained with the large image databases ImageNet and Places365. In the case of the second method we used GoogleNet CNN while in the case of the third and fourth methods we used ResNet-101, and, NasNet-Large, respectivel,. As a brief presentation of the three CNNs used, GoogleNet contains 22 layers [26], ResNet-101 contains 101 layers [26], while NasNet-Large contains 1244 layers, being part of automated machine learning (AML) [27]. The CNN models were fine-tuned by using transfer learning in order to distinguish cancerous lesions from benign lesions. We retrained GoogleNet, ResNet-101 and NasNet-Large with images extracted from the ISIC 2019 and PH^2^ databases. The algorithms were developed using MATLAB that puts at our disposal the deep learning toolbox models for GoogleNet, ResNet-101 and NasNet-Large networks. In the case of the PH^2^ database, in order to test the algorithms, we used 100 images divided into training (70 images) and validation (30 images) sets. 

Using the deep learning toolbox models for GoogleNet, ResNet-101 and NasNet-Large, networks we loaded the pre-trained networks. Then, the MATLAB function “analyze Network” was used in order to see the networks architecture and some other details regarding the network layers [26]. Thus, we noticed that for all the CNNs, excepting NasNet-Large, the first network layer, namely, the input layer, requires images of 224 × 224 × 3 size, while in case of NasNet-Large, the input layer requires images of 331 × 331 × 3 size. Therefore, our algorithms need an image resizing step. 

In order to classify the images, the final classification layer and the last learnable layer use features that are extracted by the convolutional layers. In all three networks, the two last layers, contain the details on how to combine the features into loss value, class probabilities and predicted label. To retrain the pre-trained networks to be able to classify new images, the last two layers were replaced with new layers that fitted to our images. The MATLAB function “findLayersToReplace” is then used to find the layer names and to replace them. The last layer which has learnable weights is replaced with a new two outputs layer that is fully connected. In order to set the output classes (common nevus and melanoma) automatically on the training time, we used the MATLAB function “trainNetwork”. Having new layers with a faster learning rate than the one in the transferred layers was one of our goals and this is why, the learning rate factors, “WeightLearnRateFactor” and “BiasLearnRateFactor” where increased [26]. 

We don’t want to update the parameters of previous layers because this process will be time consuming and thus, in the next step, the learning rates of previous layers are set to zero in order to “freeze” them. By freezing the previous layers, we avoid any overfitting. 

Next step is the network training. As mentioned above, the input layer of the networks requires a certain size of the images. Therefore, we used an augmented image datastore in order to resize the training images automatically. Using data augmentation, we also avoid the overfitting of the network and the extra details storage of the images. In the case of validation images, we also used an image datastore but without data augmentation. MATLAB gives us the opportunity to change some training options by using “trainingOptions” function. Parameters such as “InitialLearnRate”, “MiniBatchSize” and “MaxEpochs” are set. The initial learning rate was set to a lower value in order to slow down the training in the layers that were not yet frozen. The maximum number of epochs was then set. In case of transfer learning, we don’t need to perform the learning process for many epochs. Then, by setting the mini batch size, we update the weights and we evaluate gradient of loss function [26]. The calculation of the classification accuracy and the validation images classification using the fine-tuned networks, represent the last step. 

**Figure 6:**
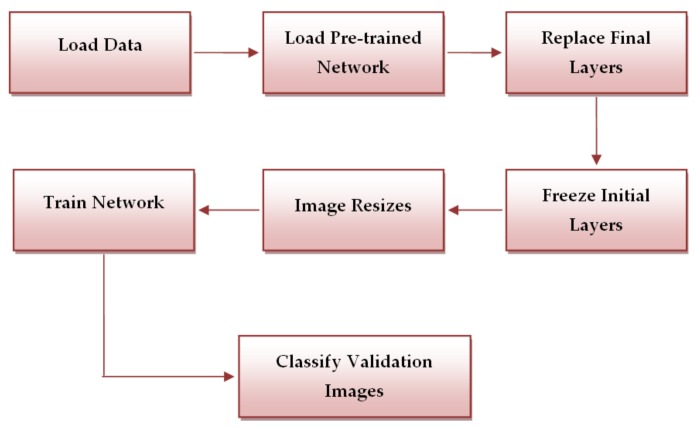
Workflow of CNN-based systems.

The workflow of the above-mentioned systems is presented in Figure 6.

### 2.4. Methodology for Skin Lesion Detection Using Feature – Based Methods 

The system consists of two major components: one is learning and other is validation. The algorithm was implemented in MATLAB. The steps of the system are as follows:

The first step of the preprocessing component is to convert the image from RGB to grayscale that is usually enough to distinguish edges. Another fact is that the comparison in grayscale requires simple scalar algebraic operators and we don’t need to differentiate colors [28]. The second step of the preprocessing component is to resize the image to classify to have the same dimension as the images used in the learning step to avoid errors in matrix operations. The last step in the preprocessing component is to highlight the region of interest (lesion) by binarizing the image. In order to obtain the binarized image we used an adaptive threshold matrix. 

By using an adaptive threshold, the image is splited into certain frames and for each frame a threshold is calculated. The adapttresh MATLAB function helped us to calculate a locally adaptive threshold. Then, we used the imbinarize function to convert the intensity image to a binary one. The obtained result is a matrix with the same size as the gray scaled image that consists of normalized intensity values in the [0,1] range [26]. 

**Figure 7:**
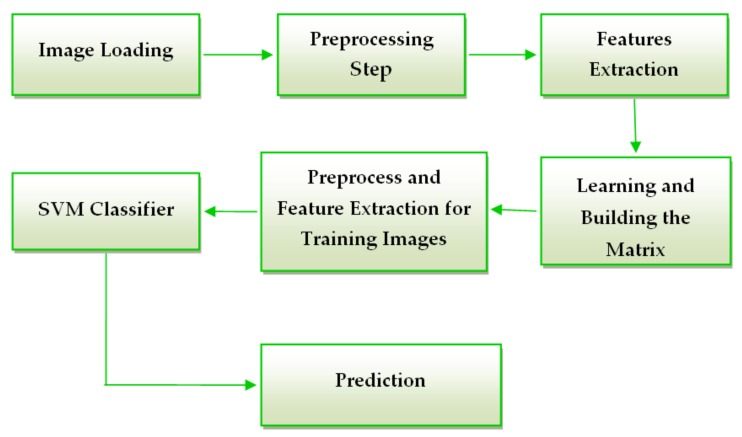
Flow chart of the proposed systems.

According to Figure 7 the next step of the skin lesion detection system must be the feature extraction. In this study we chose to use histogram of oriented gradient (HOG) features. The idea behind the HOG descriptor is that the appearance and shape of an object found in an image can be described by the edge directions or by the distribution of intensity gradients. The image is split into small connected area called cells, and a HOG direction is formed for the pixels that are placed in each cell. The concatenation of these histograms forms the descriptor [29]. 

After feature extraction, the classifier is trained by the help of the learning matrix. The learning matrix is formed by the HOG feature vectors extracted from the training images. It is very important to be sure that the HOG feature vector encodes the correct amount of information about the object. The ExtractHOGFeatures MATLAB function helps us not just to extract the HOG features, but also to display a window that shows us what is the meaning of “correct amount of information”. Modifying the size of the HOG cell, we saw the important effect that these parameters have on the quantity of shape information in the feature vector [26]. A cell size of [8 × 8] didn’t encode sufficiently the shape information, while a cell size of [2 × 2] encoded too much shape information and increased the dimensionality of the HOG feature vector significantly. The best shape information was achieved by using a 4 × 4 cell size. The spatial information that was encoded by this size setting was enough in order to visually identify a skin lesion shape.

For the classification step the algorithm uses a SVM classifier. A support vector machine can be used for classification, regression and more other tasks by creating one or more hyper planes in an infinite-dimensional space [30]. The best separation is achieved when the hyper plane has the biggest distance to the nearest training point of one of the classes. The classifier generalization error decreases as the larger the edge is [31]. The function used in MATLAB is fitcecoc. This function gives a trained, multiclass, error-correcting output codes (ECOC) model by using the training matrix and the class labels [26]. In our case, the labels are the name of each image.

### 2.5. Methodology for Skin Lesion Detection Using the Global Decision System

**Figure 8:**
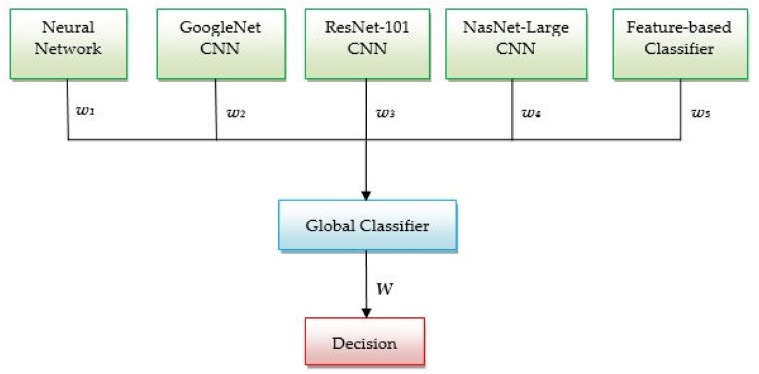
Architecture of global decision system *w_1_*, *w_2_*, *w_3_*, *w_4_*, and *w_5_* are the weights_;_ *W* is the global index of decision.

As mentioned in the introduction section, the main purpose of this study is to develop a global decision system that combines all above mentioned methods. Thus, we propose the system architecture, based on decision fusion, presented in Figure 8. The idea behind this system is that combining more efficient classifiers in one system we can achieve a highest accuracy for the skin lesion classification. Depending on the accuracy obtained in the validation phase, each method offers to the global classifier a result with a certain degree of confidence. A global index of decision *W* is calculated considering the weights and, also, the individual decisions associated to each classifier (*w_1_*, *w_2_*, *w_3_*, *w_4_*, *w_5_*, and, respectively, *d_1_*, *d_2_*, *d_3_*, *d_4_*, *d_5_*) as in Equation (2):

(2) $$W=\overset{5}{\underset{i=1}{\sum }}w_{i}d_{i}$$

The weight *w_i_* is equal to the accuracy calculated in the validation phase for each classifier (four neural networks and a feature-based classifier). These weights can take values between 0 and 1, corresponding to the mentioned classifier accuracies. The individual decision *d_i_* equals 1 if the corresponding classifier indicates melanoma (Me) and 0 if it didn’t indicate a melanoma (in this case, a common nevus (Cn)). The global decision that the investigated lesion is a melanoma is taken if threshold condition (3) occurs:

(3) $$W\geq 0.7\cdot W_{max},$$

where *W*_max_ corresponds to a unanimous decision (4):

(4) $$W_{max}=\overset{5}{\underset{i=1}{\sum }}w_{i}$$

The factor 0.7 was chosen experimentally.

## 3. Experimental Results

As a brief recapitulation, to training, validation, and testing the proposed algorithms, we used two skin lesion image databases (PH^2^ and ISIC 2019) and 300 images. Because the PH^2^ database has only 40 images with melanoma, the number of images used from the two databases is unbalanced, 1:2 (Table 1). From the table it can be seen that about 70% of images were used for learning and 30% for validation.

Next, the experimental results of our proposed algorithms on both databases are presented. For each individual classifier and each database confusion matrix is calculated. The confusion matrix for the validation phase will be associated with a weight for fusion in the global classifier. 

### 3.1. Experimental Results for Skin Lesion Detection Using Neural Network 

**Figure 9:**
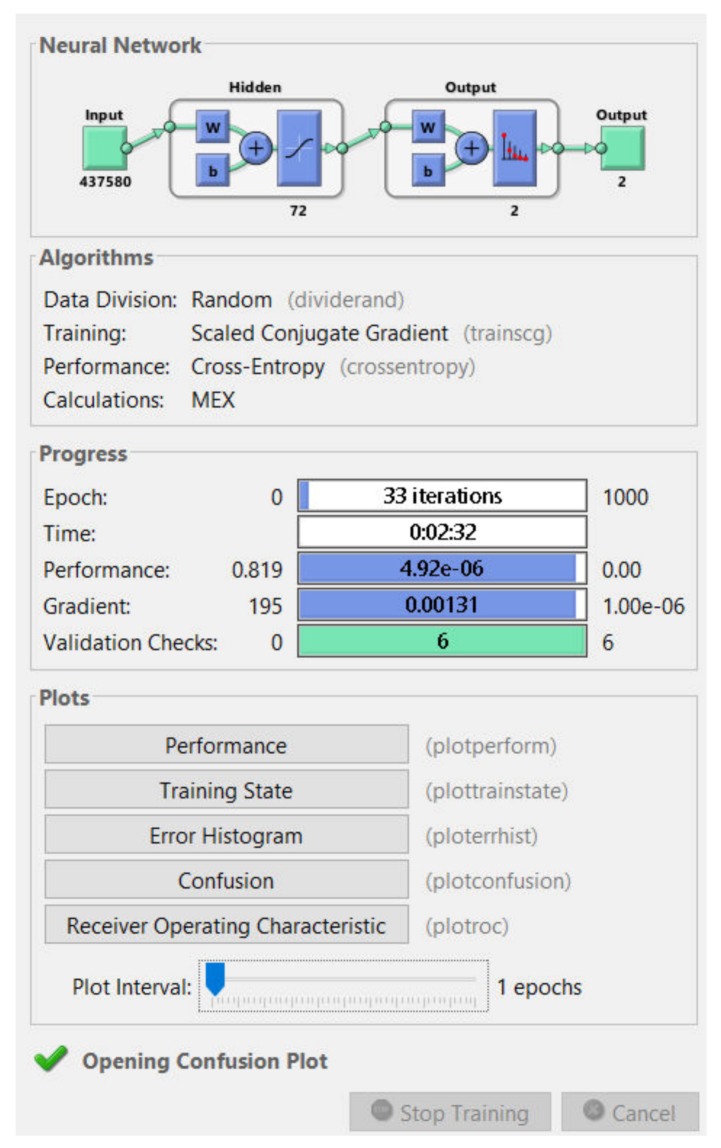
Neural network training tool.

First, we tested the algorithm using PH^2^ database. Several attempts were made until we got the best result of classification. We created a neural network made of two targets (common nevi and melanoma) in the output layer and 72 hidden layers (Figure 9). This combination of layers gave the best skin lesion classification results.

**Figure 10:**
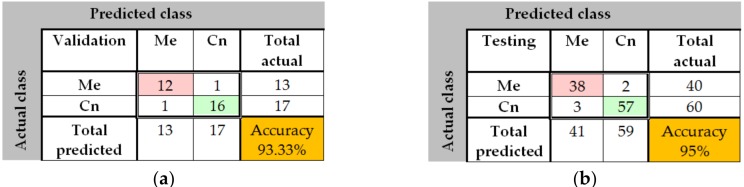
Confusion matrices in case of PH^2^ database (Me – melanoma, Cn – common nevus): (**a**) Validation phase; (**b**) Testing phase.

The training process was analyzed by using the MATLAB neural network toolbox. The training accuracy and information about the algorithms and status can be observed by the help of the neural network training tool. The training process achieved a few 33 epochs, while the time elapsed was just 2 min and 32 s. Due to helpful MATLAB toolbox, we also could analyze the confusion matrix, by seeing useful plots. The confusion matrices (Figure 10) are computed for the validation phase (30 images) and for the testing phase (100 images). The predicted classes are represented as columns while the actual classes are represented as rows. The correct classified images are represented on the first diagonal of the matrix while the incorrect are represented on the second diagonal of the matrix. In the bottom right is presented the accuracy, calculated as in Table 2. In case of validation phase, one common nevus (Cn) was bad classified as melanoma (Me) and one Me was classified as Cn. The same observation is for testing: 2 Me was classified as Cn and 3 Cn was classified as Me. By analyzing this result, we can say that the neural network with 72 hidden layers could be a good choice for distinguishing the malign lesion from benign lesion. In order to have better results we need more training images. 

**Figure 11:**
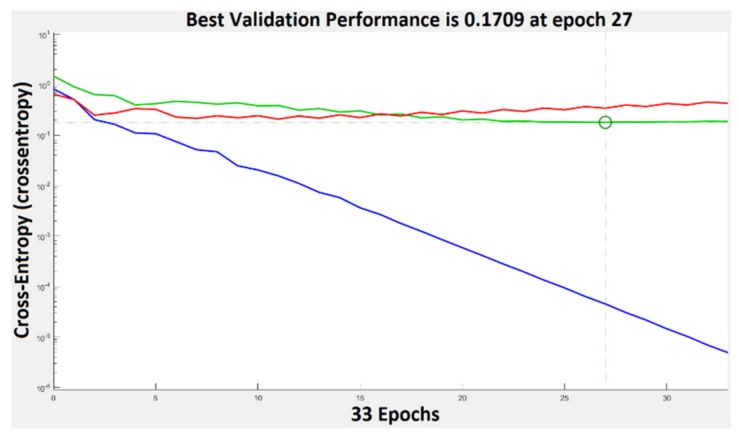
Training performance in case of PH^2^ database. Blue represents the training, green—the validation, red—the testing, and circle—the best validation performance.

The performance plot is a useful plot that helps us to observe how the network means square error decreases rapidly as it learns. The best validation performance was 0.1789 at epoch 27. This can be seen in Figure 11. If the number of epochs increases, the blue line will indicate a smaller error on the training data. The validation error is represented by a green line. In our case the performance of the trained network with learning data is better than with data that are not implicated in the learning process and this is because the training curve decreases more than the validation curve. When the validation error stops, decreasing the training stops. The error on the data used for testing the training is represented by the red line. We can’t say that we have the best training process because the error increases. 

**Figure 12:**
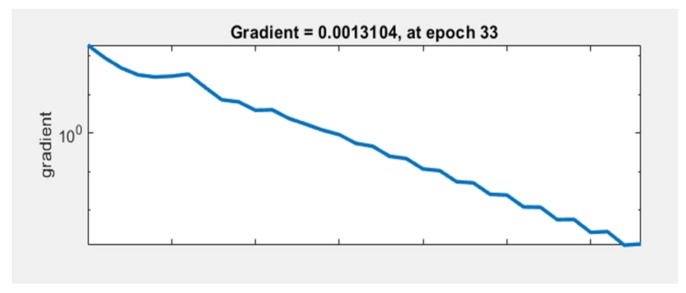
Neural network train state plot.

The graph in Figure 12 represents the gradient value at each iteration. The network performance increases as the gradient value is closer to 0. In our case, the lowest gradient value is 0.0013104 at epoch 33. 

**Figure 13:**
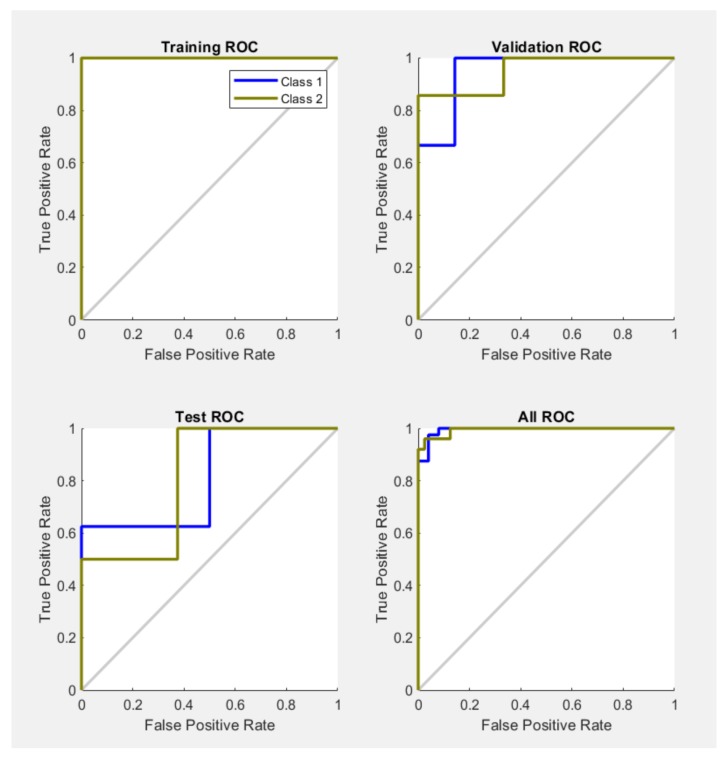
Neural network training, testing and validation states ROC plot.

The results of the proposed network simulation are also presented with the help of the receiver operating characteristic (ROC) curve. The measure of validity of a diagnostic test is associated with the area that is placed under the ROC curve. The ROC graphs for, testing, training and validation of the system are presented in Figure 13. The overall ROC of the system can be observed in the last graph.

If we had a test without any errors the specificity and sensitivity should be 100%. In this case we must have a graph with points in the upper-left side. This is the case of training ROC, but not also the case of test ROC where we can see that the specificity and sensitivity for class 1 and 2 doesn’t reach very fast the value of 100%, but in most part the results are quite good. In the case of the validation ROC, the value of 100% is reached pretty fast. The performances of some tested neural networks with different numbers of hidden layers are shown in Table 1. The formulas of the performance indicators are listed in Table 2, where *TP* is true positive (melanoma image correctly identified as melanoma), *TN* is true negative (common nevus correctly identified as common nevus), *FP* is false positive (common nevus incorrectly identified as melanoma), and *FN* is false negative (melanoma incorrectly identified as common nevus).

**Figure 14:**
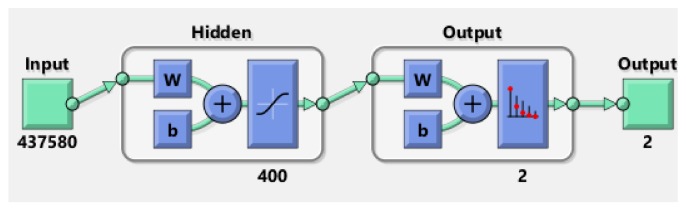
Neural Network architecture for ISIC 2019 database.

In the case of ISIC 2019 database, having much more images than PH^2^ database, the obtained results where much weaker. After several attempts, the best result was obtained by a neural network having not less than 400 hidden layers. The elapsed time was 150 min. The network (Figure 14) was also analysed by the confusion matrix and the training performance.

**Figure 15:**
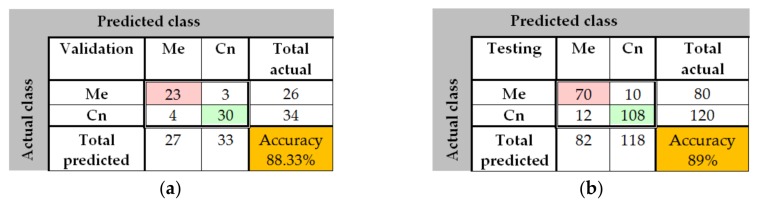
Confusion matrices for ISIC 2019 database (Me—melanoma, Cn—common nevus): (**a**). Validation phase, (**b**). Testing phase.

**Figure 16:**
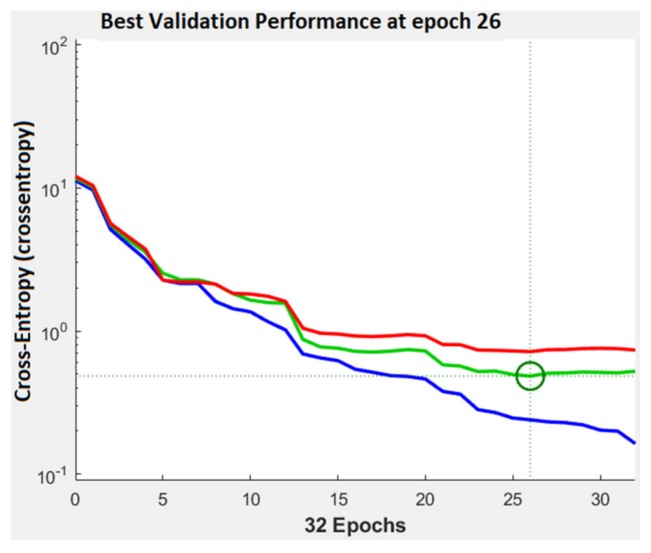
Training performance in case of ISIC 2019 database. Blue represents the training, green—the validation, red—the testing, and circle—the best validation performance.

In Figure 15, the confusion matrix for the validation phase shows an accuracy of 88.33%. 23 images where well classified as Me, 30 images where correct classified as Cn, 4 Cn images where misclassified as Me, and 4 of Me images where badly classified as Cn. In the case of testing confusion matrix, an accuracy of 89% was achieved. 70 images where correctly classified as Me, 108 images where correctly classified as Cn, 10 Me and 12 Cn where incorrectly classified. As seen in the performance plot (Figure 16), the best validation performance was 0.484 at the epoch 26, a bigger value than in case of PH^2^ database.

As in the case of the PH^2^ database, we tested the performance of different networks with different number of hidden layers also for ISIC 2019 database. The best results are obtained for the Neural Network with 400 layers. Table 3 presents a brief summary of the classification performances for the two databases.

### 3.2. Experimental Results for Skin Lesion Detection Using Convolutional Neural Networks

In this subsection we analyze the behavior of three CNNs (GoogleNet, ResNet-101, and NasNet-Large) as individual classifiers for melanoma detection. From each database 70% of images were used for training and 30% of the images where used for validation (in both PH^2^ and ISIC 2019). Just like we did above, we will present first the results obtained on PH^2^ database and after that we will present the results obtained on ISIC 2019. 

**Figure 17:**
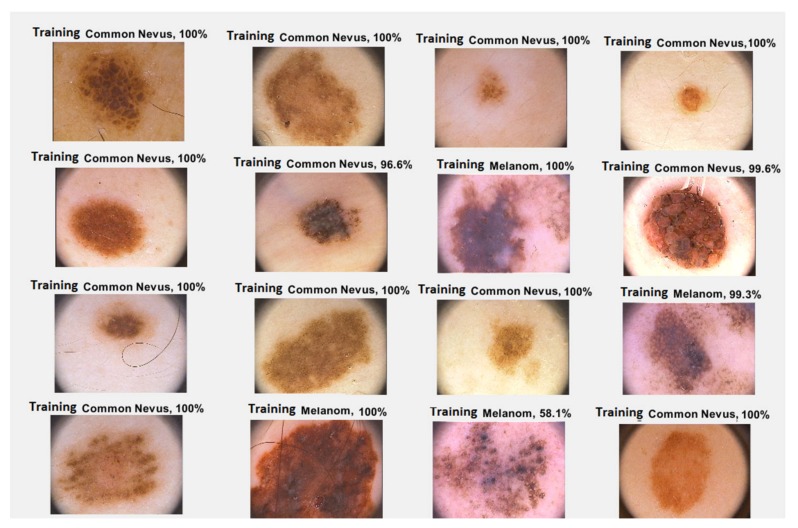
Validation images with predicted labels and predicted probabilities in case of GoogleNet.

**Figure 18:**
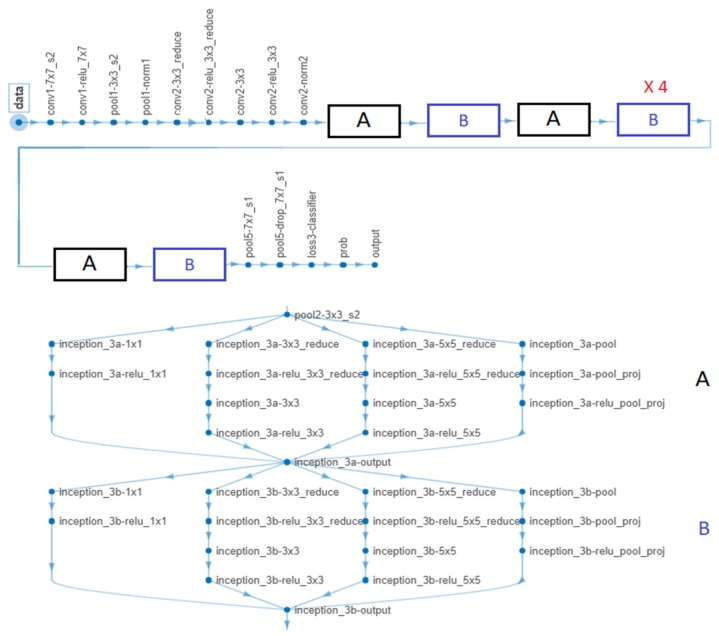
GoogleNet CNN architecture obtained by the help of MATLAB.

**Figure 19:**
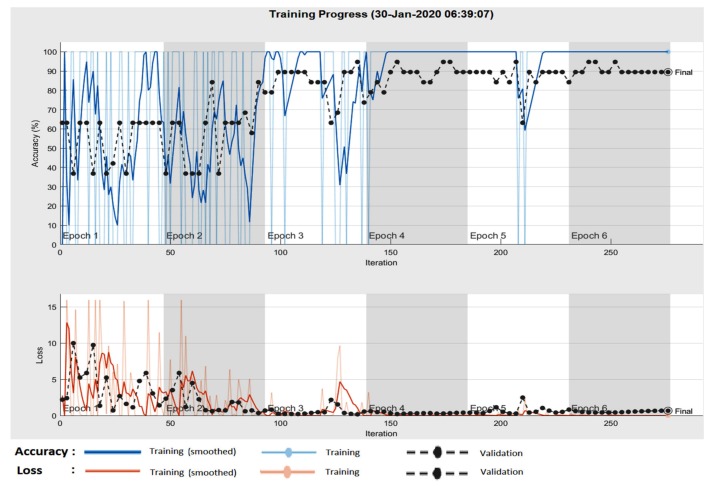
Training progress GoogleNet CNN pre-trained with the Places 365 image database in the case of PH^2^.

**Figure 20:**
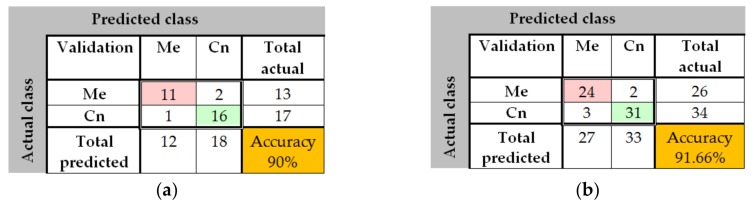
(**a**) Confusion matrix for validation phase in the case of GoogleNet pre-trained on Places365 for the PH^2^ database; (**b**) Confusion matrix for validation phase in the case of GoogleNet pre-trained on Places365 for the ISIC 2019 database.

In the case of GoogleNet CNN, pre-trained with the Places 365 database and applied to both databases, the best results can be seen in Figure 17, Figure 18, Figure 19, Figure 20a,b. In this case, we set a mini batch size of 1 while the weight learning rate factor and the bias learn rate factor was set to 20. Thus, in Figure 17, there are presented several sample validations images. For each of them we presented the predicted labels and the predicted probabilities of the images having those labels. The GoogleNet architecture in Matlab implementation is presented in Figure 18 and contains several similar modules (A and B). The GoogleNet CNN pretrained with Places 365 database gives the best results (Figure 19). As can be seen from the confusion matrix (Figure 20a) the validation accuracy was 90%. This accuracy will be the associated weight for GoogleNet classifier in the global system based on decision fusion. The time elapsed was 1 min and 45 s. The number of epochs was 6. The confusion matrix presented in Figure 20a also shows the type of images where the network was wrong. 

In the case of GoogleNet applied on ISIC 2019, the best result was also obtained when pretraining the CNN with Places 365 image database. In this case, the minibatch, the weight learning rate factor and the bias learning rate factor were set to 10. An accuracy of 91.66% was achieved in 1 min and 46 s as seen in Figure 20b. 

**Figure 21:**
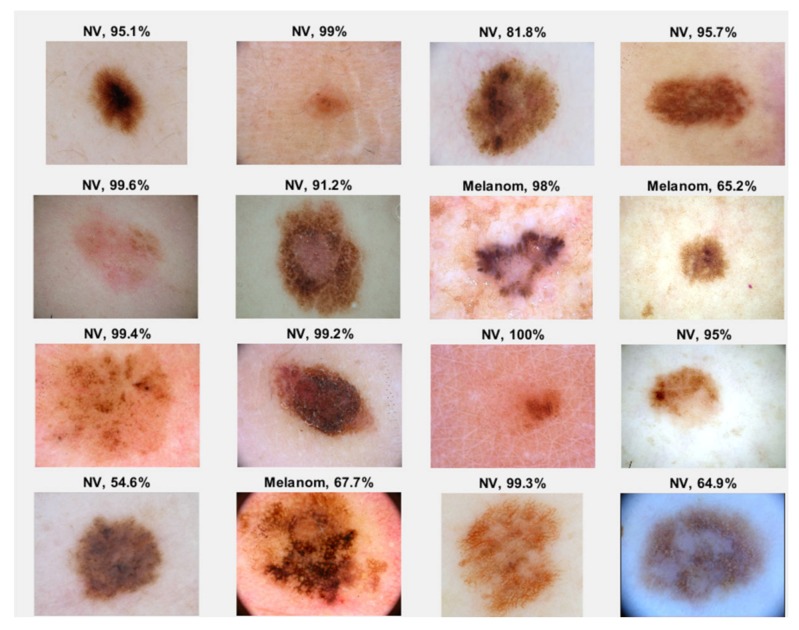
Validation images with predicted labels and predicted probabilities in the case of ResNet-101.

**Figure 22:**
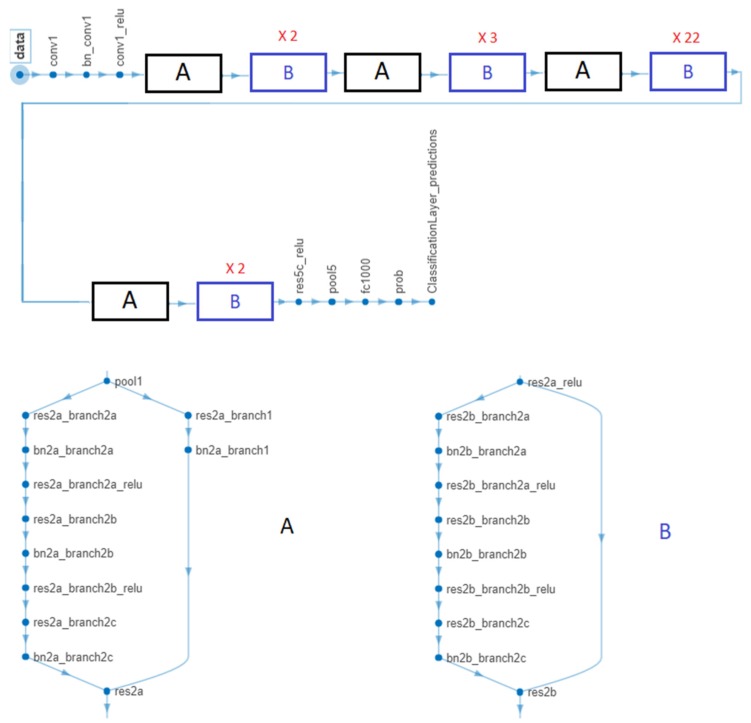
ResNet-101 CNN architecture obtained by the help of MATLAB.

**Figure 23:**
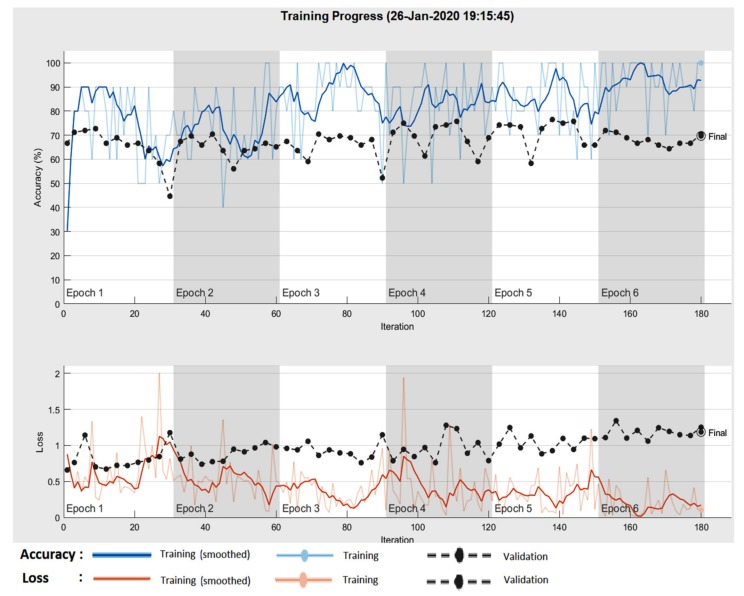
Training progress ResNet-101 CNN.

**Figure 24:**
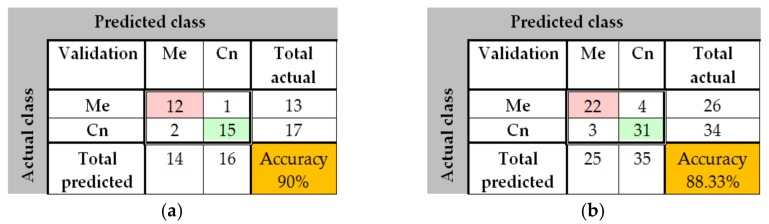
(**a**) Confusion matrix for validation phase in the case of ResNet-101 CNN for the PH^2^ database; (**b**) Confusion matrix for validation phase in the case of ResNet-101 CNN for the ISIC 2019 database.

The results obtained in the case of ResNet-101 CNN can be seen in Figure 21, Figure 22, Figure 23, Figure 24. Thus, in Figure 21 we displayed some validation images with predicted labels and the associated probabilities of the images having those labels. In this case, we set a mini batch size, a weight learning rate factor and a bias learn rate factor of 10. The ResNet-101 CNN architecture is presented in Figure 22 and similar modules A and B can be observed. The training progress during epochs is detailed in Figure 23. As seen from confusion matrix (Figure 24a) the obtained validation accuracy was 90%. The elapsed time was 6 min and 7 s. The number of epochs was 6. The confusion matrix shown in Figure 24b corresponds to ISIC 2019 dataset indicates an accuracy of 88.33% and shows that 3 cases of Cn where misclassified as Me and other 4 Me images where misclassified as Cn. 

**Figure 25:**
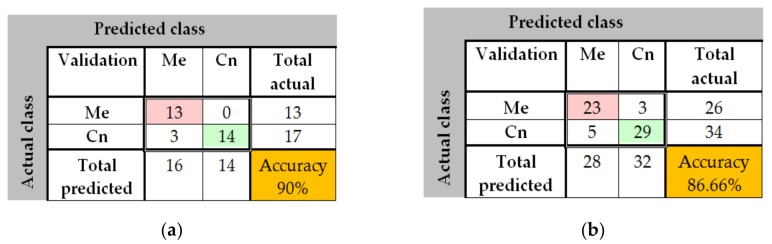
(**a**) Confusion matrix for validation phase in the case of NasNet-Large CNN for the PH^2^ database; (b) Confusion matrix for validation phase in the case of NasNet-Large CNN for the ISIC 2019 database.

**Figure 26:**
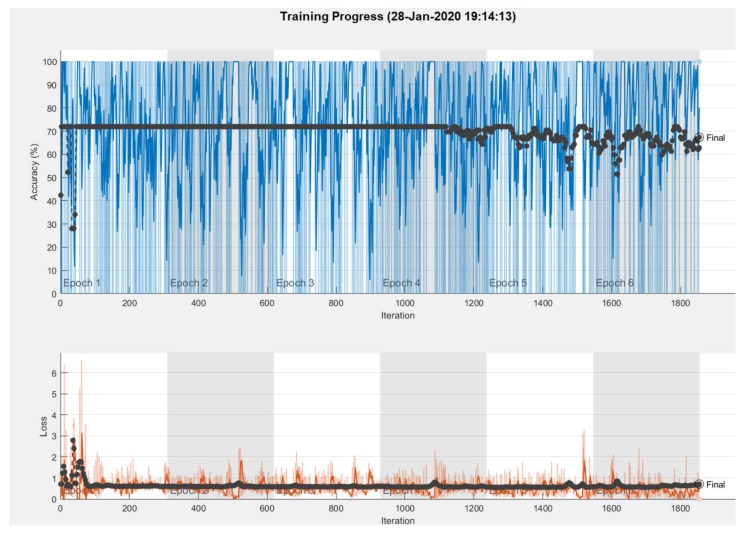
Training progress NasNet-Large CNN.

**Figure 27:**
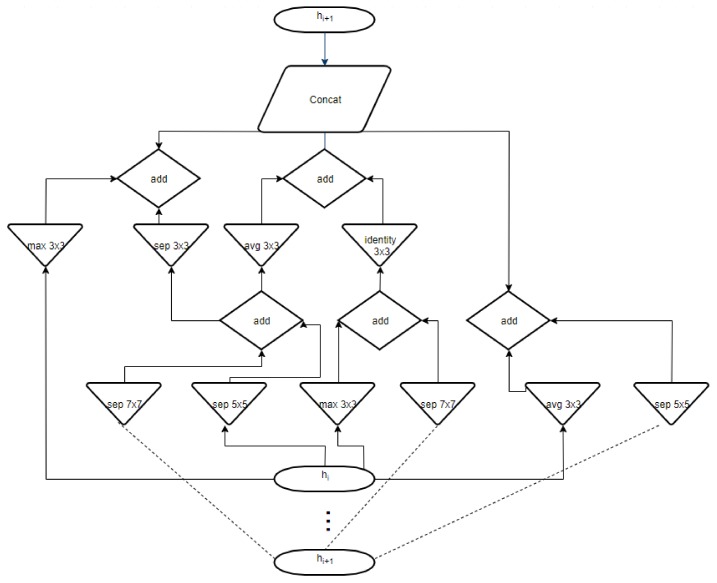
NasNet-Large CNN architecture.

The results obtained in the case of NasNet-Large CNN can be seen in Figure 25, Figure 26, Figure 27. As shown in Figure 25a from confusion matrix (validation phase), for the PH^2^ dataset an accuracy of 90% was obtained. Similarly, for the ISIC 2019 database, an accuracy of 88.66% was obtained (see confusion matrix from Figure 25b. The training progress of this CNN can be observed from the diagrams in Figure 26. The time required by the system in order to deliver this result was 748 min and 59 s. The maximum iteration number was 1854. Finally, the architecture of the NasNet-Large CNN is presented in Figure 27.

### 3.3. Experimental Results for Skin Lesion Detection Using Feature-Based Method 

**Figure 28:**
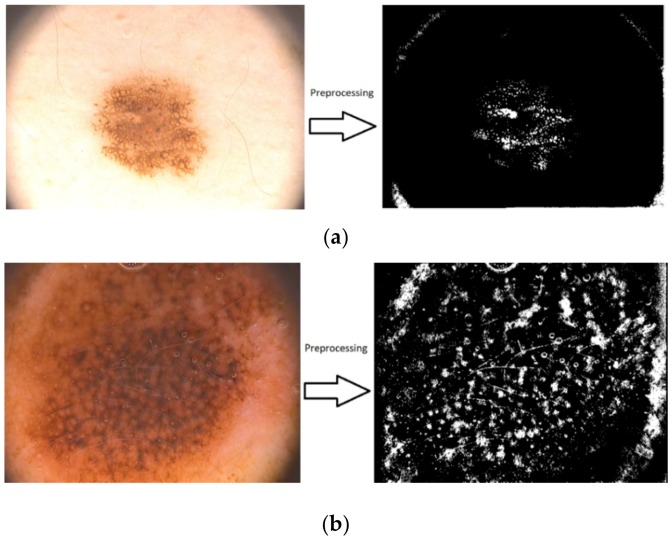
Sample images showing the results of preprocessing component: (**a**) common nevus and (**b**) melanoma.

**Figure 29:**
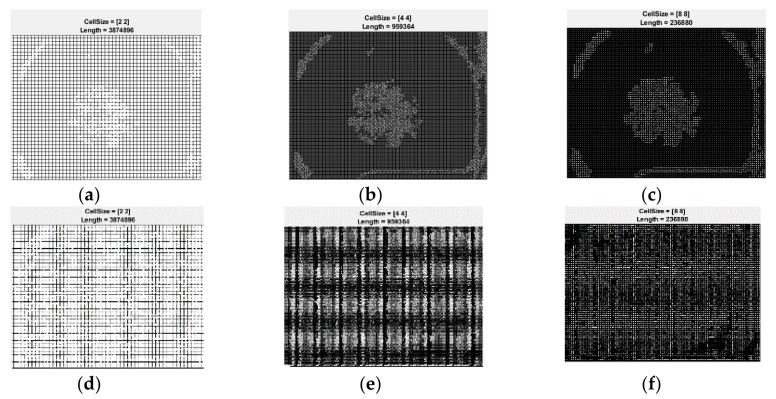
Sample images showing the effect of the variation of HOG cell size parameter has on the amount of shape information encoded in the feature vector. (**a**)Cell size 2 × 2—common nevus, (**b**)Cell size 4 × 4—common nevus, (**c**) Cell size 8 × 8—common nevus, (**d**) Cell size 8 × 8—melanoma, (**e**) Cell size 4 × 4—melanoma, and (**f**) Cell size 8 × 8—melanoma.

**Figure 30:**
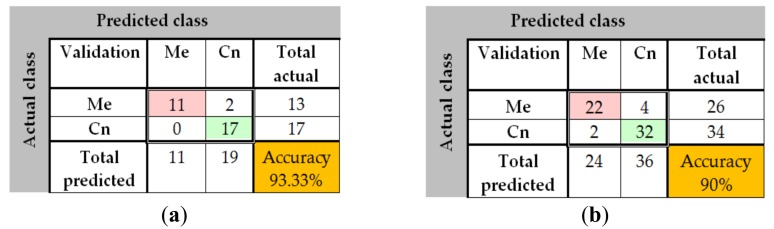
(**a**) Confusion matrix for validation phase in the case of Feature-based method for PH^2^ database; (**b**) Confusion matrix for validation phase in the case of Feature-based method for ISIC 2019 database.

The same sets of images as in the first methods were used for testing and validation of the feature-based methods. The results are shown in Figure 28a,b, Figure 29a–f and Figure 30a,b. In Figure 28 the results of the preprocessing step which consists onf RGB to grayscale conversion and image binarization on a common nevus image (a) and a melanoma image (b) can be seen. As mentioned above, after the preprocessing step, the next step is the feature extraction. In Figure 29 the reason why we mentioned in Section 2.4 that the best shape information was achieved by using a 4×4 cell size can be seen. Thus, a cell size of 8×8 can’t encode enough shape information, while a cell size of 2 × 2 encoded too much shape information. As shown in Figure 30a for PH^2^ dataset an accuracy of 93.33% was obtained. Similarly, for the ISIC 2019 database, an accuracy of 90% was obtained (Figure 30b).

### 3.4. Experimental Results for Skin Lesion Detection Using the Global Classifier

From he taccuracies calculated to validation of individual classifiers, the associated weights were established (Table 4) from Equations (3) and (4). Although there are different thresholds for the two databases, as can be easily observed, when the factor 0.7 was chosen experimentally, a common threshold can be used for the two databases (for example, 3.15).

The proposed global classifier showed high accuracy when applying in both the PH^2^ and ISIC 2019 databases. In Table 5 the obtained performance results can be seen. The number of images used to test the algorithm was 100 from PH^2^ (40 Me and 60 Cn) and 200 from ISIC 2019 (80 Me and 120 Cn).

## 4. Discussion

In this paper, we have presented the advantages of the decision fusion method considering the accuracy attributed to different neural networks-based and feature-based methods for skin lesion detection. More exactly, we combined all these methods to obtain a global decision system with higher accuracy than each individual classifier. First, we developed a new neural network using MATLAB functions and toolboxes. The sensitivity, specificity, accuracy, and DCS of the NN indicate good performances for both databases (PH^2^ and ISIC 2019). The NN was configured and trained separately for the mentioned databases to obtain the best results (72 hidden layers for the PH^2^ database and 400 hidden layers for the ISIC 2019 database). Second, we detected skin lesions using CNNs which were pre-trained with the large image database ImageNet and Places 365. The CNNs were then fine-tuned in order to classify skin lesions by using transfer learning. We retrained GoogleNet, ResNet-101, and NasNet-Large with the same images that we used in the first case. The best results were obtained using GoogleNet pre-trained with Places 365 image database. Finally, we used a featured-based method in order to detect the skin lesions. The method used the support vector machine algorithm. The learning phase was done using HOG features. In order to highlight the skin lesion shape, we used an adaptive thresholding. It can be observed that the individual classifiers have different accuracy for the two databases and, generally, the results for PH^2^ database are better than for ISIC 2019.

By combining all these methods in a single decision-fusion system based on individual decisions and the associated weights, we obtained results with higher accuracy. In the case of the PH^2^ database, an accuracy of 95% was achieved, while in case of the ISIC 2019 one, an accuracy of 93% was achieved.

To achieve better results, we pre-processed all images to remove the hair from the images. In this case, we used DullRazor software because is simpler than others. Not in all cases the removal of hair was succeesful. This is because there were images in which thick dark hair was covering the entire lesion and after applying the algorithm, there were some remaining signs on the location where the hair was removed. Also, very thin hairs couldn’t be removed by this algorithm. Another problem that where raised by image quality was the presence of some marks drawn by dermatologists near the lesions and, also, the presence of water drops. 

Over the years, numerous studies on this subject thave been conducted. Below (Table 6) we present a comparison between the results of our proposed system and the results obtained by other authors.

We can see that our result (95%) obtained on the PH^2^ database is better than the one obtained in [12] (81% accuracy), which used the same database. The closest result of the one that we obtained was reported by Codella et al. [6]. Greater accuracy values were obtained for the segmentation process of lesions without classification. We believe that we could improve our system by applying more filters on the preprocessing step to remove the image noise.

## 5. Conclusions

This paper proposed a new system based on artificial intelligence for melanoma detection considering the decision fusion of five classifiers, one of them also created by the authors (a neural network). The rest contains three CNN type and one SVM—features type classifiers. The individual classifiers and the global classifier were training on two well-known databases (PH^2^ and ISIC 2019) with different results concerning the accuracy. For both databases the accuracy was higher than that of the individual classifiers (more than 1.66% for PH^2^ and 1.33% for ISIC) or other methods proposed in the cited references. As feature work, we want to create a large database with better images by applying some preprocessing filters. We should also take care about the details of the patients, like age, sex, skin color, and so on. 

## Figures and Tables

**Table 1:** The number of images used for experimental results (Me – melanoma, Cn – common nevi).

Database	Learning			Validation			Testing		
	Me	Cn	Total	Me	Cn	Total	Me	Cn	Total
Images PH^2^	27	43	70	13	17	30	40	60	100
Images ISIC 2019	54	86	140	26	34	60	80	120	200

**Table 2:** Expressions for the performance indicators.

Performance Indicators	Formula
Specificity	$Specificity=\frac{TN}{TN+FP}$
Senitivity	$Sensitivity=\frac{TP}{TP+FN}$
Accuracy	$Accuracy=\frac{TP+TN}{TP+TN+FP+FN}$
Dice Similarity Coefficient (DSC)	$DSC=\frac{2TP}{2TP+FP+FN}$

**Table 3:** Classification performance of the proposed neural network in the validation phase.

Database	Neural Network Layers	Accuracy	Specificity	Sensitivity	DSC
PH^2^	72	93.33%	94.12%	92.31%	92.31%
ISIC 2019	400	88.33%	88.24%	88.46%	86.79%

**Table 4:** Establishing the weights of the individual classifiers and decision threshold.

Weights	*w*_1_	*w*_2_	*w*_3_	*w*_4_	*w*_5_	*W*_max_	Threshold 0.7 *W*_max_
PH^2^_Values	0.93	0.9	0.9	0.9	0.93	4.56	3.19
ISIC_Values	0.88	0.92	0.88	0.87	0.9	4.45	3.12

**Table 5:** Experimental results for the global classifier.

Database	Proposed Algorithms	Accuracy	Specificity	Sensitivity	DSC
PH^2^	Global Classifier	95%	96.66%	92.5%	93.67%
ISIC 2019	Global Classifier	93%	93.33%	92.5%	91.36%

**Table 6:** Comparison results of accuracy.

References	Method	Accuracy	Observations
[6]	Deep learning, sparse coding, SVM	93.1%	lesion classification
[9]	Linear classifier CNN deep learning	85.5%	lesion classification
[10]	CNN	98.5%	only segmentation
[11]	Dense deconvolutional network	93.9%	only segmentation
[12]	Feature-based method	81%	lesion classification
[16]	Two deep learning method	91.2%	lesion classification
[32]	Decision support based on color and texture	81%	lesion classification
[Our]	Decision fusion, CNN, feature-based method	95% / 93%	lesion classification
